# Parenting boys with conduct problems and callous-unemotional traits: parent and child perspectives

**DOI:** 10.1007/s00787-022-02109-0

**Published:** 2022-11-14

**Authors:** Ruth Roberts, Eamon McCrory, Helene Joffe, Harriet Phillips, Anne Gaule, Essi Viding

**Affiliations:** 1 Division of Psychology and Language Sciences, University College London, London, UK; 2 Anna Freud National Centre for Children and Families, London, UK

**Keywords:** Callous-unemotional traits, Conduct problems, Parenting, Qualitative methods

## Abstract

Parenting children with conduct problems (CP) is challenging, yet very little research has examined parenting using both quantitative and qualitative methods, from the perspective of the child and their parent/caregiver, and separately for those with high vs. low levels of callous-unemotional traits (HCU vs. LCU). One hundred and forty-six boys aged 11–16 [Typically developing (TD) *n *= 31; CP/HCU *n* = 35; CP/LCU *n* = 35] and their parents/caregivers completed the Alabama Parenting Questionnaire and provided a written qualitative statement describing their respective experiences of parenting/being parented. Parents/caregivers of CP/HCU boys reported more difficulty with child monitoring and supervision than parents of TD boys. This was echoed in qualitative reports of parents of CP/HCU boys reporting concerns regarding their child’s safety. Parents/caregivers of both groups of CP boys reported more inconsistent discipline than parents of TD boys. Parental qualitative descriptions of challenging behavior in CP/HCU boys, and difficulties with setting boundaries and motivating CP/LCU boys, provided further insight to the potential triggers for inconsistent discipline. Qualitative reports from boys with CP indicated that they understood the parenting challenges their parents/caregivers faced. These findings replicate and extend previous work on the associations between parenting and CP. Children with CP/HCU and CP/LCU show some commonalities and differences in their parenting experiences and CP children and their parents/caregivers do not necessarily share all the same perceptions or concerns. CP interventions often involve parent/family engagement and this research highlights the continued importance of examining both parent and child perspectives.

**Supplementary Information:**

The online version contains supplementary material available at 10.1007/s00787-022-02109-0.

## Introduction

Parenting is thought to play a crucial role in socialization and in the development of guilt and empathy, and has received considerable attention as a risk factor for conduct problems (CP) and callous-unemotional (CU) traits [1–5]. Negative (e.g., harsh, coercive, inconsistent) aspects of parenting have been associated with increased CP symptoms, whilst positive (e.g., warm, sensitive, consistent) aspects of parenting have been associated with decreased CP symptoms [6–8]. Although less work has focused on the relationship between parenting and CU traits, there is now an accumulating evidence base suggesting that the associations are similar to those seen regarding CP [2, 6–14]. However, only a handful of studies have reported parenting practices in subgroups of children with CP, contrasting those with high vs. low levels of CU traits (CP/HCU vs. CP/LCU). In a seminal study, Wootton et al. [15] investigated the degree to which negative parenting is associated with the levels of CP among children with HCU vs. those with LCU. Although both groups reported elevated levels of negative parenting, only the CP/LCU group showed a dose–response relationship between the degree of negative parenting and CP. More recent studies have found that children with CP/HCU are more likely to experience harsh parenting in early childhood than those with CP/LCU [16] and have parents who are less able to monitor their whereabouts during adolescence than parents of adolescents with CP/LCU [17].

Prior findings relating to parenting and CP, CU, and CP/HCU vs. CP/LCU groups likely reflect both the competencies of parents as well as the challenges introduced by a child who may be provocative and more difficult to parent consistently [18, 19]. In line with this notion, longitudinal research has found that children with CP evoke more harsh and negative parenting [20] and that elevated CU traits in children are associated with increased parent-reported inconsistent discipline, decreased parental involvement, increased corporal punishment, and high levels of parental distress [10, 19, 21].

While quantitative measures of parenting can chart the extent of negative and positive parenting practices, they are less able to capture some of the context surrounding parenting experience. Qualitative studies on parenting children with CP are relatively scarce but have the potential to elucidate the nature of the parenting experience in ways that are not captured by traditional questionnaire methods. These include specific difficulties in managing the child’s behavior, nuanced descriptions of parental emotional responses to the child’s behavior, and the impact of child’s behavior on family and community relationships [22, 23]. To our knowledge, no studies have collected qualitative data to examine differences in parenting between CP/HCU and CP/LCU children, although one recent study has focused on qualitative descriptions of parental perceptions of the child in these groups [24]. This study revealed differences in how parents of children with CP/HCU and CP/LCU viewed their child, with parents of children with CP/LCU reporting better rapport with their children—which may have relevance for parenting practices. Given the heterogeneous nature of CP behaviors, qualitative data may help to elucidate the complexity of daily parenting struggles encountered by families with a child with CP.

### The current study

The aim of the current study was to advance our understanding of parenting boys with CP/HCU and CP/LCU, exploring both the experiences of parents/caregivers and the children themselves, using both quantitative and qualitative methods. The use of multiple measures can help identify areas of difficulty when parenting a child with CP and varying levels of CU traits. In addition, such an approach helps develop a more nuanced understanding of the practical and subjective nature of those difficulties. For example, parents of children with CP often feel blamed for their child’s behavior [25] and can feel that their considerable parenting challenges and efforts to help their child are not acknowledged. There has been a limited amount of literature focusing on such challenges, in the context of parenting a child with CP with high vs. low levels of CU traits. To examine possible differences in parenting experiences in families of CP/HCU, CP/LCU and TD boys, this study assessed both parent/caregiver and child reports on five domains of parenting as measured by the Alabama Parenting Questionnaire (APQ) [26]: involvement, positive parenting, poor monitoring/supervision, inconsistent discipline, and corporal punishment. In line with the prior findings [10, 19, 21], we predicted that the CP/HCU group would report reduced parental involvement (parent and child-reported) and parents/caregivers of children with CP/HCU would report difficulty with child monitoring and supervision. Based on prior findings in relation to CP and CU traits [6, 7], we predicted that parents/caregivers of both CP/HCU and CP/LCU children would report more inconsistent discipline and less positive parenting than parents of TD children. To explore parenting experiences not readily captured by questionnaire measures, this study also employed a structured qualitative approach, with written qualitative descriptions of parenting provided by parents/caregivers of CP/HCU and CP/LCU boys, and descriptions of being parented provided by the boys themselves.

## Methods

### Participants

One hundred and forty-six boys aged 11–16 years and their parent/caregiver were recruited to take part in the current study via newspaper advertisements and through engagement with mainstream and alternative provision schools who serve children with behavioral difficulties in the greater London area. University College London Research Ethics Committee (Project ID number: 0622/001) gave approval for the research protocol. Detailed information sheets describing the aims and participation in the study were provided to parents/caregivers and children and written informed consent/assent was obtained. Study exclusion criteria for child participants included a diagnosis of autism spectrum disorder, any reported neurological abnormality, and/or a score of < 70 on a standardized cognitive assessment. No exclusion criteria were applied for parents. Details of participant characteristics are displayed in Table 1.

**Table 1:** Demographic data

	TD controls (*n* = 45)			CP/LCU (*n* = 57)			CP/HCU (*n* = 44)			*p* value^a^	Post hoc*
Characteristics and questionnaires	Mean	S.D	Min–Max	Mean	S.D	Min–Max	Mean	S.D	Min–Max		
Child age (years)^b^	14.48	1.63	11.42–16.91	14.68	1.59	11.10–16.99	14.90	1.40	11.23–16.77	0.454	
Child IQ (full score, two-subtest WASI)^c^	97.02	12.94	72–122	97.09	15.14	70–129	93.10	11.63	77–117	0.304	
Child ethnicity^b,f^	6:27:12			7:31:19			7:28:9			0.711	
SES^b^	2.80	1.17	1–5.50	3.03	1.27	1–5.00	3.51	1.18	1.5–5.50	0.015	1 > 3
ICU^d^	24.27	6.98	7.00–40.00	33.57	7.78	10.00–43.00	50.09	5.49	45.00–69.00	0.000	1 < 2 < 3
CASI conduct disorder^d^	0.69	0.73	0.00–2.00	6.47	3.53	3.00–22.00	11.54	5.36	4.00–25.00	0.000	1 < 2 < 3
CASI attention deficit hyperactivity disorder^e^	11.35	7.26	1.00–38.00	22.01	11.77	2.00–46.59	26.89	12.81	4.00–53.00	0.000	1 < 2/3
CASI generalized anxiety disorder^e^	4.88	3.77	1.00–19.00	8.10	4.52	1.00–21.00	9.68	4.48	1.00–19.00	0.000	1 < 2/3
CASI major depressive episode^e^	3.31	2.12	2.00–13.00	6.20	4.55	2.00–19.00	7.39	3.95	2.00–17.00	0.000	1 < 2/3
Child alcohol use^c^	0.70	1.76	0.00–7.00	2.68	4.56	0.00–19.00	2.38	3.87	0.00–18.00	0.021	1 < 2
Child drug use^c^	0.18	0.83	0.00–4.00	1.91	3.43	0.00–11.00	2.72	5.08	0.00–22.00	0.003	1 < 2/3
Parent self-report psychopathy^e^	6.26	9.47	0.00–39.74	6.29	8.50	0.00–34.00	9.00	10.59	0.00–0.42	0.298	
Parent/caregiver informant^e,g^	44:1:0			54:1:2			40:2:2			0.828	
Number of parents/caregivers^b,h^	25:20			30:27			16:28			0.144	
Child birth order^e,i^	20:13:5:6			23:19:6:8			16:16:8:4			0.567	
Number of people living in household^e,j^	7:11:15:7:4			3:14:26:10:4			10:10:11:8:5			0.434	

*TD* typically developing, *CP/LCU* conduct problems and low levels of callous-unemotional traits, *CP/HCU* conduct problems and high levels of callous-unemotional traits, *S.D*. standard deviation, *WASI* Weschler Abbreviated Scale of Intelligence, *SES* socio-economic status, *ICU* Inventory of Callous-Unemotional Traits, *CASI* Child and Adolescent Symptom Inventory. *p<0.05, Tukey post hoc comparison

^a^All *p* values obtained using ANOVA, except child ethnicity and number of parents/caregivers (Chi-square) and parent/caregiver informant, birth order, and number of people living in the household (Fisher’s exact)

^b^Measures obtained at screening phase, parent report

^c^Measures obtained at testing session, child report

^d^Measures obtained at screening phase, parent and teacher report

^e^Measures obtained at testing session, parent report

^f^Counts for each ethnicity (Black:White:Mixed/Other)

^g^Counts for each rater category [Mother:Father:Other (Foster/Adoptive/Grandparent)]

^h^Counts for two-parent/carer household:single-parent/carer household

^i^Counts for 1st:2nd:3rd:4th+ born

^j^Counts for family size of 2:3:4:5:6+ family members

Children with CP were assigned to CP/HCU and CP/LCU groups using a median split (CP/HCU > 43 and CP/LCU ≤ 43) on the combined parent and teacher scores on the *Inventory of Callous-Unemotional Traits* (ICU; Essau et al., 2006) [27]. See Online Resource 1 for details regarding group assignment and the median split approach.

#### Additional child and parent measures

The *Wechsler Abbreviated Scale of Intelligence* (WASI) [28] was used to assess child cognitive ability. Substance use in boys was assessed via the self-report *Alcohol Use Disorder Identification Test* (AUDIT) [29] and the self-report *Drug Use Disorder Identification Test* (DUDIT) [30]. The *Child and Adolescent Symptom Inventory* (CASI-4R) [31] scales for conduct disorder (CD), attention deficit hyperactivity disorder (ADHD), generalized anxiety disorder (GAD) and major depressive episode (MDE) were completed by parents/caregivers to assess for conditions that are commonly comorbid with CP. Parents/caregivers provided information about parental education and employment to ascertain socio-economic status (SES) and completed the *Self-Report Psychopathy Short Form* (SRF-SF) [32] to assess parent/caregiver psychopathy. In this sample, Cronbach’s alpha for the SRF-SF was 0.83. Parents also provided details about child birth order, number of parents/caregivers in the household (biological, stepparent, foster and adoptive parents, grandparents), and the total number of people living in the household.

#### Alabama Parenting Questionnaire (APQ) [26]

The APQ assesses five dimensions of parenting commonly associated with CP: involvement, positive parenting, poor monitoring/supervision, inconsistent discipline, and corporal punishment (further details of the subscales are found in Online Resource 2). Parent/caregivers and children completed the 42-item parent and child forms respectively, rating frequency of parenting behavior on a five-point scale (1 = never to 5 = always). Cronbach’s alpha for parent APQ subscales in this sample were as follows: involvement 0.75, positive parenting 0.77, poor monitoring/supervision 0.76, inconsistent discipline 0.77, and corporal punishment 0.68. Cronbach’s alpha for child APQ subscales in this sample were as follows: involvement with mother 0.79, involvement with father 0.93, positive parenting 0.85, poor monitoring/supervision 0.75, inconsistent discipline 0.66, and corporal punishment 0.55. All subscales were in the acceptable range except for the child corporal punishment scale which reflects the 3-item length of this scale.

#### Qualitative descriptions of parenting: parental description of challenges of parenting; child description of being parented

Parents/caregivers were asked to describe their parenting experiences with written responses to the following question: *What are the biggest challenges in parenting your child?* Boys were asked to provide a written response to the following open-ended question: *Please think of the person who is most involved in taking care of you. Can you tell us a little about how (or the way) they take care of you?*

Full details of the qualitative method, study procedure, and analysis protocols are provided in Online Resource 3.

## Results

### Demographic characteristics

There were no significant group differences on child age, child IQ, child ethnicity, number of parent/caregivers, parent/caregiver informant, birth order, total number of people living at home, and parental psychopathy. The groups differed on SES, with the TD group having significantly higher SES than the CP/HCU group, *F* (2,143) = 4.332, *p* = 0.015; no other group differences were found. The two CP groups were higher on ADHD, *F* (2,144) = 23.556, *p* < 0.0001, generalized anxiety, *F* (2,144) = 14.531, *p* < 0.0001, major depression, *F* (2,142) = 13.962, *p* < 0.0001, and child drug use, *F* (2,143) = 6.020, *p* = 0.003 use as compared to the TD group; no other significant group differences were found on these variables. The CP/LCU group reported higher alcohol use than the TD group, *F* (2,142) = 3.971, *p* = 0.021; no other group differences in alcohol use reached significance.

### APQ parent report

There was a group difference on parent-reported poor monitoring and supervision, *F* (2,143) = 5.044, *p* = 0.008, (TD *M* = 9.12; CP/LCU *M* = 11.34; CP/HCU *M* = 12.99). Pairwise group comparisons revealed significant differences between TD and CP/HCU groups with a medium effect size (*p* = 0.005; *d* = 0.72). No other significant group differences were found.

There was also a group difference on parent-reported inconsistent discipline, *F* (2,143) = 6.783, *p* = 0.002, (TD *M* = 7.75; CP/LCU *M* = 10.26; CP/HCU *M* = 10.18). Pairwise group comparisons revealed significant differences between TD and CP/HCU groups with a medium effect size (*p* = 0.007; *d* = 0.67) and TD and CP/LCU groups with a medium effect size (*p* = 0.003; *d* = 0.68). The CP/HCU and CP/LCU groups did not differ significantly from each other.

No group differences emerged on parent-reported involvement, *F* (2,143) = 0.623, *p* = 0.538, positive parenting, *F* (2,143) = 0.915, *p* = 0.403, or corporal punishment, *F* (2,143) = 2.252, *p* = 0.109.

#### Covariate analysis

The effect of group on parent-reported poor monitoring and supervision was no longer significant after adjusting for AUDIT, *F* (2,125) = 1.405, *p* = 0.249.

The effect of group on parent-reported inconsistent discipline remained significant after adjusting for SES, ADHD, GAD, MDE, AUDIT and DUDIT, *F* (2,125) = 4.806, *p* = 0.010 (TD / CP/LCU *d* = 0.605; TD / CP/HCU *d* = 0.650).

### APQ child report

There was a group difference on the child-reported involvement with father subscale, *F* (2,143) = 3.473, *p* = 0.034, (TD *M* = 23.24; CP/LCU *M* = 19.60; CP/HCU *M* = 16.95). Pairwise group comparisons revealed significant differences between TD and CP/HCU groups with a medium effect size (*p* = 0.026; *d* = 0.56). There was no significant difference between TD and CP/LCU groups or the two CP groups on the involvement with father subscale.

There was a trend-level difference in child-reported inconsistent discipline by group, *F* (2,143) = 2.904, *p* = 0.058 (two tailed), (TD *M* = 7.38; CP/LCU *M* = 8.91; CP/HCU *M* = 9.30). Because our predictions were one tailed, we conducted pairwise group comparisons which showed the difference between TD and CP/HCU groups had a large effect size (*p* = 0.066; *d* = 1.18). There was no significant difference between TD and CP/LCU groups or the two CP groups on the inconsistent discipline subscale.

The groups did not differ on child-reported involvement with mother, *F* (2,141) = 2.092, *p* = 0.127, positive parenting, *F* (2,141) = 0.055, *p* = 0.947, poor monitoring and supervision, *F* (2,143) = 1.168, *p* = 0.314, or corporal punishment, *F* (2,142) = 1.632, *p* = 0.199.

#### Covariate analysis

The effect of group on child-reported father involvement was no longer significant after adjusting for SES and MDE, *F* (2,125) = 0.625, *p* = 0.537.

### Post hoc analyses on parent/caregiver–child agreement on APQ ratings

Because partially different patterns of findings emerged in parent and child APQ analyses, we ran post hoc intra-class correlation (ICC) analyses for all the APQ scales that were comparable between parents/caregivers and children (i.e., all except parental involvement, which was assessed separately with regard to mothers and fathers in the child APQ) in the CP groups. These analyses showed moderate agreement between parents/caregivers and children (ICC range = 0.44–0.68). These analyses suggest that although parent/caregiver and child assessments of parenting variables relate to each other meaningfully, they are not identical and likely explain why some differences emerge in the group analyses of parenting.

### Qualitative findings

#### Challenges of parenting CP/HCU and CP/LCU children

Parents/caregivers of CP/HCU boys described grave concerns for their child’s safety, with one parent describing it as *‘my biggest fear’*. Concerns over safety were described by parents/caregivers of CP/HCU boys both in terms of difficulties in monitoring their child’s whereabouts and ‘*keeping him safe and off the streets*’, as well as, problematic peer affiliations, which one parent described as, *‘worry over his safety and peer pressure to engage in unsociable behavior or illegal activity’*.

Parents/caregivers of CP/HCU boys described challenges with extreme child behavior including, *‘violence’* and *‘aggression’*, and, *‘the unpredictable outbursts which can escalate in seconds’*. CP/HCU parents also described fatigue and stress from parenting their child. One CP/HCU parent described the last six years as being an *‘emotional and stressful time… this was very hard for the whole family, especially me’.*

Parents of CP/LCU boys, on the other hand, described difficulties with parental influence, including challenges in motivating their child, *‘trying to persuade him to do something he doesn’t want to do’,* as well as, difficulties with enforcing rules and boundaries, *‘will not confirm or follow a routine… cannot follow one instruction’,* and, *‘instilling a stronger sense of discipline’*.

#### CP/HCU and CP/LCU boys’ description of being parented

Both CP/HCU and CP/LCU boys described parental support, with CP/HCU boys describing parental willingness to support in the face of adversity, with one CP/HCU boy reporting *‘it didn’t matter how I treated her, she was always nice to me’*. CP/LCU boys, on the other hand, described support in terms of parental understanding, ‘*she understands me and now I realize how well she has raised me’*, and guidance, *‘If I do something wrong, I am usually spoken to; If I do something right I am praised’*.

A considerable number of CP/HCU (31%) and CP/LCU (22%) boys described the experience of being parented solely in terms of provision of basic needs with no mention of any emotional support or affection, *‘she gives me food, she dresses me, she pays for my house bills’*. The absence of emotional descriptions occurred very infrequently in TD boys’ descriptions of caregiving (8%).

A full description of extracted themes and supporting quotations from parents/caregivers and children are included in Online Resource 4.

## Discussion

In line with our predictions, parents/caregivers of CP/HCU boys reported more challenges with monitoring and supervision of their child (as measured by the APQ), than parents/caregivers of CP/LCU and TD boys. This finding was supported by qualitative analysis, with parents/caregivers of CP/HCU boys reporting serious concerns for their child’s safety owing to difficulties with monitoring and with peer affiliations that parents/caregivers perceived as problematic. As predicted, parents/caregivers of both CP/HCU and CP/LCU boys reported challenges with inconsistent discipline (as measured by the APQ) compared with TD parents. Qualitative reports detailed concerns regarding management of violent and aggressive behavior in CP/HCU boys and difficulty with exerting parental influence on CP/LCU boys. These findings help to provide a more nuanced picture of varied challenges of managing different subgroups of children with CP. Contrary to our predictions, parents/caregivers of CP/HCU and CP/LCU boys did not differ from parents/caregivers of TD boys on positive parenting (as measured by the APQ). However, as expected, boys with CP/HCU reported less involvement with their fathers (as measured by the APQ), than CP/LCU and TD boys, although this finding was not corroborated by parent/caregiver ratings. This may reflect the fact that parent/caregiver ratings were predominantly provided by mothers rather than fathers. CP/HCU and CP/LCU boys described qualitatively different experiences of support from parents/caregivers, with CP/HCU boys describing support from parents/caregivers even in times when it was not necessarily warranted, and CP/LCU boys describing parental understanding and guidance. Both groups of CP boys tended to qualitatively describe their parent solely as a provider of basic needs.

The finding that parents/caregivers of CP/HCU boys differed significantly from parents/caregivers of TD boys on the monitoring and supervision subscale of the APQ is broadly in line with previous research which found that parents/caregivers of CP/HCU children reported reduced knowledge (as assessed by the monitoring/supervision subscale of the APQ) and reduced monitoring (as assessed by a questionnaire measuring parental control and solicitation) of their child over time [17]. The effect of group on parent-reported poor monitoring and supervision was no longer significant after adjusting for child alcohol use. It is not surprising that these variables would be associated with each other, as presumably less effective parental monitoring would yield more opportunities for child alcohol use and therefore adjusting for alcohol use in the analysis will have likely removed variance shared with parental monitoring. Qualitative descriptions of the challenges of parenting shed more nuanced light on the difficulties in monitoring and supervising a child with CP/HCU. Parents/caregivers of CP/HCU boys reported challenges in knowing their child’s whereabouts and in keeping their child off the streets, which caused them to have great concern for their child’s safety. Parents/caregivers of CP/HCU boys also qualitatively described the need to monitor who their child was associating with, as peers were thought to be exerting a negative influence on the child. The current pattern of findings is also likely to, in part, reflect the fact that children with higher levels of CU traits engage in more severe and premeditated antisocial acts, which is perhaps not surprising given their diminished capacity for empathy, guilt and social affiliation [33]. Parents/caregivers of CP/LCU boys were not significantly different from parents/caregivers of CP/HCU or TD boys in monitoring and supervising their child as measured by the APQ. In other words, the CP/LCU group appeared to score somewhere in between the CP/HCU and TD groups in this domain of parenting. In contrast with the parents/caregivers of CP/HCU boys, the parents/caregivers of CP/LCU boys did not qualitatively report any grave concerns about their child’s whereabouts or safety or with monitoring their child’s peer group.

Parents/caregivers of both CP/HCU and CP/LCU boys differed significantly from parents/caregivers of TD boys on the inconsistent discipline subscale of the APQ. Previous research has found that inconsistent discipline was associated with increases in CU [10] and also related to CP [34] and the current findings suggest that inconsistent discipline is a challenge for parents/caregivers of both CP/HCU and CP/LCU boys. Qualitative reports from parents/caregivers helped to elucidate possible reasons why it may be difficult to consistently enforce rules and discipline both groups of CP boys. Parents/caregivers of CP/HCU boys reported challenges with extreme behavior which caused considerable stress and exhaustion for both the parents/caregivers and the family. It is not hard to imagine how parents/caregivers of CP/HCU boys may be wary of provoking a violent outburst when attempting to discipline their child or may choose to ignore less serious offences in an effort to not disturb the peace. This is consistent with previous research which found parents/caregivers’ qualitative descriptions of CP/HCU boys as being unpredictable when provoked [24]. Parents/caregivers of CP/LCU boys, on the other hand, reported challenges in maintaining boundaries and motivating their child. Although parents/caregivers of CP/LCU boys did not report the same ‘wear and tear’ from the continuous battles with their child, one can imagine it being dispiriting to have a child who refuses to follow rules or get things done, and this might contribute to lapses in discipline. The effect of group remained significant after controlling for various CP comorbidities. The robustness of this finding, along with the qualitative descriptions, indicates that it is very difficult to consistently discipline boys with CP.

There were no group differences in both parent and child-reported positive parenting subscales of the APQ, which was surprising given the challenges associated with CP and CU. Qualitative reports of parenting were in line with the APQ findings, with parents/caregivers and children in all groups describing positive aspects of parenting. Both groups of CP parents/caregivers qualitatively reported being very invested in their child’s well-being. CP/LCU boys demonstrated a recognition that their parents/caregivers understood them and were trying to provide guidance in their qualitative descriptions of being parented, with boys reporting that parents/caregivers corrected poor behavior and rewarded positive behavior. Additionally, CP/HCU boys qualitatively described support from parents/caregivers even when their behavior was causing a considerable amount of wear and tear on the family.

CP/HCU boys reported lower involvement with fathers as compared to CP/LCU and TD children. This is consistent with previous research which found that children with high levels of CU traits reported reduced levels of parental involvement [21]. The effect of group was no longer significant after controlling for SES and MDE. Future research will want to examine the impact of reduced involvement on CP/HCU boys in greater depth.

Interestingly, boys in both CP/HCU and CP/LCU groups showed an increased tendency, as compared to TD boys, to qualitatively describe their parents solely as providers of the basic necessities of life. This suggests that some boys with CP see the relationship with their parent/caregiver as transactional in nature, and that they are not registering any emotional content (descriptions of love or affection) in the way they spontaneously think about their experience of being parented. This is consistent with findings from Dadds et al. [35] who reported that children with CP/LCU report less reciprocal affection with their mothers than TD children, and that this is even further reduced in CP/HCU children (despite their mothers being similar to control mothers on expressions of affection). There may be unmeasured individual differences in the parent–child dyad that are contributing to the reasons why some CP boys are not mentioning emotional support and/or affection when describing their experience of being parented and this warrants further investigation. This novel finding highlights the importance of examining the experience of being parented from the child’s perspective, and in their own words.

This study has limitations which should be noted when interpreting the findings. Parents/caregivers in this study were predominantly mothers or female caregivers and this study focused on parenting in boys given the higher prevalence of CP in males. Future research may want to examine both quantitative and qualitative experiences of parenting, with mothers and fathers (including male and female caregivers) and both boys and girls with CP to elucidate how parenting experiences may differ by sex. It is worth noting that families traveled to a university in central London to take part in a research study and will likely not include parents and children with the most significant clinical impairment. Finally, although this study focused on both quantitative and qualitative examination of parenting, future studies, especially of younger children, may consider use of an objectively coded observational assessment of parenting.

The strengths of the current study include the examination of both parent and child perspectives on parenting, which highlighted where parenting is having an impact even if it is not outwardly apparent in the child’s behavior. For example, parents/caregivers of CP/LCU boys described challenges in motivating and setting boundaries, which may be contributing to inconsistency in disciplining their child. However, CP/LCU boys’ qualitative descriptions of being parented suggested that they are registering their parents/caregivers’ efforts in supporting them and guiding them, despite their challenging behavior. Qualitative reports of parenting also highlight possible areas for intervention. For example, some CP boys’ descriptions of being parented suggested that they do not always consider an emotional connection with parents/caregivers which could be a possible area for relationship building. Clinicians may want to consider the context around the parenting behavior and both child and parent/caregiver’s perspectives to be able to provide appropriate support.

## Conclusions

Parents/caregivers regularly experience blame and stigma relating to their perceived inability to control their child’s CP behavior [25], but too often, the nature of the parenting challenge, and how this relates to parenting behavior, is not considered. The current study found that parents/caregivers of CP children had concerns for their child’s well-being, and provided a more nuanced understanding of the challenges of parenting a child with CP—including how parenting experiences differ for CP/HCU and CP/LCU groups. The current study also highlighted the value in gathering child perspectives of parenting experience, both in terms of understanding parental impact and informing intervention efforts. Families of children with CP are often marginalized and would benefit from advocacy and understanding of the difficulties they face.

### Supplementary Information

Below is the link to the electronic supplementary material.

Supplementary file1 (DOCX 17 KB)

Supplementary file2 (DOCX 14 KB)

Supplementary file3 (DOCX 17 KB)

Supplementary file4 (DOCX 20 KB)

## Data Availability

The qualitative dataset generated during and/or analysed during the current study is not publicly available due to the potentially sensitive nature of the data that may enable identification of participants. For this reason, the institutional ethics committee has not granted permission to share these data. The individual level quantitative data generated during and/or analysed during the current study is not publicly available due to lack of institutional ethics committee permission to share individual level data. However, anonymised, group level data are available from the corresponding author on reasonable request.
