# Supplementary material for: Parenting boys with conduct problems and callous-unemotional traits: parent and child perspectives
Source: Eur Child Adolesc Psychiatry. 2022 Nov 14;32(12):2547–55. doi: 10.1007/s00787-022-02109-0 (PMC10682176; doi:10.1007/s00787-022-02109-0)
Supplement: Supplementary file 1 — Supplementary file1 (DOCX 17 KB) [file 787_2022_2109_MOESM1_ESM.docx]

#### Online resource 1. Group assignment and median split approach

Screening questionnaires assessing CP, CU traits, and psychopathology were completed by parents/caregivers and teachers to determine CP/HCU, CP/LCU, and TD groups prior to participation. Screening measures were scored by taking the highest ratings from either the parent or teacher questionnaire for each item^1^.

The *Child and Adolescent Symptom Inventory* (CASI-4R)^2^ Conduct Disorder scale (CASI-CD) was used to assess CP. Cut-off scores for inclusion in the CP group were as follows: parent report = ≥ 4 (ages 10–12) and ≥ 3 (ages 13–16) or teacher report = ≥ 3 (ages 10–12), ≥ 4 (ages 13–14), and ≥ 6 (ages 15–16). These scores are associated with a clinical diagnosis of CD^3^.

The *Inventory of Callous-Unemotional Traits* (ICU)^4^ was used to assess CU traits. A median split of the ICU scores for boys meeting CP criteria was used to determine assignment to CP/HCU (ICU score greater than 43) or CP/LCU groups (ICU score less than or equal to 43).

Boys in the TD control group scored less than or equal to 2 on the CASI-CD, less than 43 on the ICU and less than seventeen for total difficulties on the *Strengths and Difficulties Questionnaire* (SDQ)^5^.

We employed a median split approach to separate the children with CP to groups with high and lower levels of CU traits (HCU vs. LCU), for the following reasons: 1) Effects of CU traits do not often emerge as interactions and can instead lead to suppressor effects in correlational analyses^6^; 2) The median split approach has, in the past, successfully delineated groups of children with CP who have different cognitive-affective processing patterns. The pattern of results in these two groups has often been such that if they had been combined, researchers might have missed deficits in either group^7,8^; 3) Suppressor effects can generate difficulties for interpretation, which mean that effects of CU traits may not emerge in interactions, although the CP/HCU and CP/LCU children look very different. The group centric analyses thus make it easier to interpret the translational relevance of findings, which is more challenging when examining suppressor effects in continuous analyses, for example. It is important to note that concerns regarding loss of power from dichotomizing relate to the case of bivariate normality^9^, but using continuous measure of CP and CU can generate problems if modelled together, given the absence of bivariate normality - high CU traits almost invariably denote high levels of CP, but not the other way around^10^.

The median split of 43 is comparable with previous research which suggested that a score of 41 may represent a clinically meaningful cut-off score to identify HCU children from combined ratings^11^.

***References***

1. Piacentini, J.C., Cohen, P., Cohen, J. (1992) Combining discrepant diagnostic information from multiple sources: are complex algorithms better than simple ones?  Journal of Abnormal Child Psychology, 20*,* 51–63.
2. Gadow, K. D., & Sprafkin, J. (2009) The symptom inventories: an annotated bibliography. Stony Brook, New York: Checkmate Plus.
3. Gadow, K. D., & Sprafkin, J. (1998) Adolescent symptom inventory-4 norms manual. Stony Brook, New York: Checkmate Plus.
4. Essau, C. A., Sasagawa, S., & Frick, P. J. (2006) Callous-Unemotional Traits in a Community Sample of Adolescents. Assessment, 13(4), 454-469. https://doi.org/10.1177/1073191106287354
5. Goodman, R. (1997) The Strengths and Difficulties Questionnaire: a research note. Journal of Child Psychology and Psychiatry, and Allied Disciplines, 38(5), 581–586. Retrieved from http://www.ncbi.nlm.nih.gov/pubmed/9255702
6. Frick, P. J. (2012) Developmental Pathways to Conduct Disorder: Implications for Future Directions in Research, Assessment, and Treatment. Journal of Clinical Child and Adolescent Psychology, 41(3), 378–389. https://doi.org/10.1080/15374416.2012.664815
7. Viding, E., Sebastian, C. L., Dadds, M. R., Lockwood, P. L., Cecil, C. A. M., De Brito, S. A., & McCrory, E. J. (2012) Amygdala response to preattentive masked fear in children with conduct problems: The role of callous-unemotional traits. American Journal of Psychiatry, 169(10), 1109–1116. https://doi.org/10.1176/appi.ajp.2012.12020191
8. Schwenck, C., Mergenthaler, J., Keller, K., Zech, J., Salehi, S., Taurines, R., … Freitag, C. M. (2012) Empathy in children with autism and conduct disorder: Group-specific profiles and developmental aspects. Journal of Child Psychology and Psychiatry and Allied Disciplines, 53(6), 651–659. https://doi.org/10.1111/j.1469-7610.2011.02499.x.
9. Cohen, J. (1983) The Cost of Dichotomization. Applied Psychological Measurement, 7(3), 249–253. https://doi.org/10.1177/014662168300700301
10. Fontaine, N. M. G., McCrory, E. J. P., Boivin, M., Moffitt, T. E., & Viding, E. (2011) Predictors and Outcomes of Joint Trajectories of Callous-Unemotional Traits and Conduct Problems in Childhood. Journal of Abnormal Psychology, 120(3), 730–742. https://doi.org/10.1037/a0022620
11. Docherty, M., Boxer, P., Huesmann, L. R., O'Brien, M., & Bushman, B. (2017) Assessing callous-unemotional traits in adolescents: Determining cutoff scores for the inventory of callous and unemotional traits. Journal of Clinical Psychology, 73(3), 257-278. https://doi.org/10.1002/jclp.22313
