# Supplementary material for: Parenting boys with conduct problems and callous-unemotional traits: parent and child perspectives
Source: Eur Child Adolesc Psychiatry. 2022 Nov 14;32(12):2547–55. doi: 10.1007/s00787-022-02109-0 (PMC10682176; doi:10.1007/s00787-022-02109-0)
Supplement: Supplementary file 2 — Supplementary file2 (DOCX 14 KB) [file 787_2022_2109_MOESM2_ESM.docx]

**Online resource 2. Dimensions of the Alabama Parenting Questionnaire (APQ)^1^.**

The APQ assesses assessment of five dimensions of parenting commonly associated with CP: *involvement* (10 items; e.g. *You play games or do other fun things with your child*), *positive parenting* (6 items; e.g. *You compliment your child when he/she does well at something*), *poor monitoring/supervision* (10 items; e.g. *Your child is out with friends you don’t know*), *inconsistent discipline* (6 items; e.g. *You feel that getting your child to obey you is more trouble than it’s worth*), and *corporal punishment* (3 items, e.g. *You slap your child when he/she has done something wrong*).

***Reference***

1. Shelton, K. K., Frick, P. J., & Wootton, J. (1996) Assessment of parenting practices in families of elementary school-age children. Journal of Clinical Child Psychology, 25(3), 317–329. https://doi.org/10.1207/s15374424jccp2503_8
