# Supplementary material for: Parenting boys with conduct problems and callous-unemotional traits: parent and child perspectives
Source: Eur Child Adolesc Psychiatry. 2022 Nov 14;32(12):2547–55. doi: 10.1007/s00787-022-02109-0 (PMC10682176; doi:10.1007/s00787-022-02109-0)
Supplement: Supplementary file 3 — Supplementary file3 (DOCX 17 KB) [file 787_2022_2109_MOESM3_ESM.docx]

#### Online resource 3. Details of qualitative method, study procedure and analysis protocols

#### Qualitative method

Parents/caregivers were asked to describe their parenting experiences with written responses to the following question: *What are the biggest challenges in parenting your child?*

Boys were asked to provide a written response to the following open-ended question: *Please think of the person who is most involved in taking care of you. Can you tell us a little about how (or the way) they take care of you.* It was also ascertained who the boys described and only descriptions pertaining to parents/caregivers were included in the analyses. The majority of the boys described their mother or female caregiver (78%) and the groups did not differ significantly from each other in terms of which parent/caregiver they described (*p* = 0.761).

Parents/caregivers and children were given as much time as they needed to complete the questions and were not restricted in the length of their response. Parents/caregivers and children were advised to write what first came to their head when thinking about the question; they were made aware that there was no right or wrong way to answer the questions and there were no expectations from researchers regarding their responses. Written qualitative data collection was a preferred method of data collection for our participants as: (1) this method allows for answers to be written privately, providing increased anonymity in responding, which allowed participants the opportunity to discuss parenting experiences that may have been difficult to discuss in a face-to-face interview (2) this method allowed for participants to take their time and not feel rushed with their responses^1,2^.

***Procedure***

Parents/caregivers and children completed all assessments in a quiet testing room at University College London. Parents/caregivers and children completed the assessments separately from each other to ensure anonymity. A researcher was on hand to answer questions and offer assistance. In two cases, the parent was unable to write their response to the qualitative questions (owing to literacy problems and physical disability), so their verbal responses were written by a trained researcher. For 10 cases, boys refused to write their response to the qualitative questions (owing to literacy problems and low motivation) but agreed for the researcher to write their verbal responses for them.

***Analysis***

**Demographic characteristics.** Analysis of variance (ANOVA) was computed to explore the demographic characteristics of the groups on child age, child IQ, child substance use, child CU traits, child CP, ADHD, GAD and MDE, as well as, family SES and parental self-reported psychopathy. Where overall significant group differences were found, Tukey’s post hoc analyses were computed to examine the differences between groups. Chi-square was computed to compare groups on child ethnicity and number of parent/caregivers. Fisher’s exact test was computed to assess group differences on parent/caregiver informant, child birth order, and total number of people living at home.

**APQ.** A one-way ANOVA was computed to assess group differences on the APQ subscales. For those subscales showing overall significant group differences, Tukey’s post hoc analyses were conducted to examine the differences between groups. Effect sizes were computed to quantify the differences between groups. For those subscales showing overall significant group differences, analysis of covariance (ANCOVA) was computed to control for variables which showed group level differences, which included SES, ADHD, GAD, MDE, AUDIT and DUDIT.

Data was examined for suitability for ANOVA prior to each analysis. Boxplots were used to assess for outliers, normal distribution of data for each group was assessed by Shapiro-Wilk test, and Levene’s test was used to assess homogeneity of variance.

**Qualitative analysis of parenting descriptions.** The primary focus of this study was to understand the challenges of parenting CP/HCU and CP/LCU boys, as well as CP/HCU and CP/LCU boys’ experience of being parented, therefore TD qualitative data is not presented. All identifiable information was removed from descriptions. Data entry was checked for accuracy and completeness of statements prior to conducting analysis. Qualitative data analysis followed the six-step procedure as developed by Braun and Clarke^3^. Data for CP/HCU and CP/LCU were coded separately. Codes for CP/HCU and CP/LCU groups were clustered into overall themes by exploring the relationships between the codes and the code’s relevance to the quantitative parenting data and the child’s group assignment. The themes were examined using the data linked to each theme to ensure that the themes were supported. Themes and data for CP/HCU were compared with the themes and data for CP/LCU group and vice versa, to examine for overlap and ensure the themes were specific for each group rather than pertained to CP more generally. Data was also examined for contradictory evidence that did not support the constellation of themes for each particular group, to ensure no important information was missed and to help reduce potential bias.

To assess reliability, 15% of the qualitative transcripts were coded by a second rater. Cohen’s Kappa revealed a ‘substantial’ agreement^4^ between raters for ratings of both parent/caregiver data, К = 0.717, and child data К = 0.788. Any discrepancies were discussed and resolved between raters.

***References***

1. Edwards R, Holland J (2013) What is qualitative interviewing? Bloomsbury Academic, London.
2. Braun, V., Clarke, V., Boulton, E., Davey, L., & McEvoy, C. (2020) The online survey as a qualitative research tool. International Journal of Social Research Methodology, 24(6), 641-654. DOI: 10.1080/13645579.2020.1805550
3. Braun, V., & Clarke, V. (2006) Using thematic analysis in psychology. *Qualitative* Research in Psychology, 3(2), 77-101. https://doi.org/10.1191/1478088706qp063oa
4. Landis, J.R., Koch, G.G. (1977) Measurement of Observer Agreement for Categorical Data. Biometrics, 33, 159-174.
