# Supplementary material for: Parenting boys with conduct problems and callous-unemotional traits: parent and child perspectives
Source: Eur Child Adolesc Psychiatry. 2022 Nov 14;32(12):2547–55. doi: 10.1007/s00787-022-02109-0 (PMC10682176; doi:10.1007/s00787-022-02109-0)
Supplement: Supplementary file 4 — Supplementary file4 (DOCX 20 KB) [file 787_2022_2109_MOESM4_ESM.docx]

#### Online resource 4. Qualitative themes and supporting quotations

Qualitative themes for parents/caregivers and children are described in Table 1.

Table 1. Qualitative themes from parent/caregiver reports of challenges in parenting and child reports of being parented

| Parent / Child | Group | Theme | Codes | Example |
| --- | --- | --- | --- | --- |
| Parent |  | ***Concerns for safety*** |  |  |
|  | CP/HCUp |  | Monitoring | ‘*keeping him safe and off the streets*’ |
|  | CP/HCUp |  | Peer Influence | *‘worry over his safety and peer pressure to engage in unsociable behaviour or illegal activity’* |
|  |  |  |  |  |
|  |  | ***Behaviour*** |  |  |
|  | CP/HCUp |  | Violence and aggression | *‘the unpredictable outbursts which can escalate in seconds’* |
|  | CP/HCUp |  | Wear and tear from constant battles | *‘Everyday is hard work and a constant worry’* |
|  |  |  |  |  |
|  |  | ***Parental influence*** |  |  |
|  | CP/LCUp |  | Motivation | *‘trying to persuade him to do something he doesn’t want to do’* |
|  | CP/LCUp |  | Rules and boundaries | *‘will not confirm or follow a routine… cannot follow one instruction’* |
|  |  |  |  |  |
| Child |  | ***Support*** |  |  |
|  | CP/HCU |  | Parent willingness to support in the face of adversity | *‘If I get arrested, she’ll come get me’* |
|  | CP/LCU |  | Parental understanding | ‘*she understands me and now I realise how well she has raised me’* |
|  | CP/LCU |  | Parental guidance | *‘If I do something wrong, I am usually spoken to; If I do something right I am praised’* |
|  |  |  |  |  |
|  |  | ***Parent as a provider of basic needs*** |  |  |
|  | CP/HCU and CP/LCU |  | Basic caregiving with no mention of love or emotional connection | *‘gives me shelter, a room to sleep in’* |

***Qualitative findings***

**Challenges of parenting CP/HCU and CP/LCU children.** Themes within the parent/caregiver descriptions of the challenges of parenting are presented on the basis of their relevance to the child’s group assignment and connectedness to the APQ. Qualitative themes for parenting CP/HCU boys included concerns over child safety and child behaviour. Qualitative themes for parenting CP/LCU boys included challenges with exerting parental influence on their child.

***Concerns for safety.***

***Theme 1: monitoring****.* Keeping their children safe was a significant cause of concern for parents/caregivers of CP/HCU boys. Many CP/HCU parents/caregivers reported *‘worry’* or *‘concerns’* over the safety of their child, with one parent describing it as *‘my biggest fear’*. CP/HCU parents described a *‘lack of fear’* in their child and difficulty with *‘finding out where he is’.*  Another CP/HCU parent described the difficulty in ‘*keeping him safe and off the streets*’. One CP/HCU parent described the efforts made in monitoring her child: *‘I spend a lot of time checking up on him and driving round to make sure he is ok’.* In contrast, concern for child safety was only mentioned twice by CP/LCU parents/caregivers and was not described as a worry or concern.

***Theme 2: peer influence.*** Many CP/HCU parent/caregiver concerns over safety were connected with the child’s peer affiliations. One CP/HCU parent reported, *‘It’s hard to keep influential people away from him as he can be used and led very easily’*, and another parent described, *‘worry over his safety and peer pressure to engage in unsociable behaviour or illegal activity’*. Peer influence over CP/HCU boys’ behaviour seemed to result in CP/HCU parents having difficulty trusting their child, for example, *‘trusting him when he is out with friends’*. Parents/caregivers of CP/LCU boys did not describe challenges with the influence of peers on their child.

***Behaviour.***

***Theme 1: Violence and aggression.*** Parents/caregivers of CP/HCU boys frequently described difficulty with extreme child behaviour. Parents/caregivers described challenges with *‘aggression’*, *‘violence’*, and *‘hostility’*. One CP/HCU parent described challenges with, *‘the unpredictable outbursts which can escalate in seconds’*. Concerns over CP/HCU behaviour on others was evidenced in descriptions such as, *‘…younger sibling does not trust him when playing’*, and *‘concerns for the future as he gets bigger, for the household, and others he may form close/intimate relationships with’*. Parents/caregivers of CP/LCU boys also described challenges with their child’s behaviour but it was less extreme and more about the child exhibiting oppositional behaviours such as being *‘angry’* and *‘argumentative’*.

***Theme 2: Wear and tear from constant battles****.* Parents/caregivers of both CP/HCU and CP/LCU boys described constant battles and arguments with their children, however, CP/HCU parents described the wear and tear of parenting their child more frequently than CP/LCU parents. One CP/HCU parent reported, *‘Everyday is hard work and a constant worry’*, and another CP/HCU parent described, *‘His poor behaviour is very stressful to deal with. This can cause the whole family to be strained and unhappy’*. Another CP/HCU parent described, *‘daily battles with minor things, parenting (child) can be exhausting sometimes. He gets his money’s worth’*. CP/HCU parents also described the chronic nature of the stress on the family. One CP/HCU parent described the last six years as being an *‘emotional and stressful time… this was very hard for the whole family, especially me’*, and another parent reported, ‘*We often have arguments. It’s sometimes tiring when we keep doing this’*.

***Parental Influence.***

***Theme 1: Motivation.*** Both CP/HCU and CP/LCU parents described the need to keep their child *‘focussed’* and *‘on the right path’*, but while for CP/HCU parents this was driven by a need to keep their child safe, for CP/LCU parents this seemed to be driven by a difficulty in motivating their child. One CP/LCU parent described a challenge in, *‘helping strike the right balance between what needs to be done (duties!) and what he wants to do’*, and another CP/LCU parent described difficulty in, *‘trying to persuade him to do something he doesn’t want to do’*. CP/LCU parents described challenges in getting their child to, *‘start and finish a task’*, *‘making him achieve one task every year’*, and with their child’s *‘unwillingness to try new things’*.

***Theme 2: Rules and boundaries.*** Parents/caregivers of CP/LCU boys more frequently described considerable difficulty with children not *‘recognising’* boundaries, as well as *‘maintaining boundaries’* with their child, as compared to CP/HCU parents/caregivers. One CP/LCU parent described a challenge in, *‘instilling a stronger sense of discipline’*, and another reported that the child can be, *‘…incredibly rude and answers adults back as equals… although only 12, he will not be told what to do’*. Another CP/LCU parent described challenges when their child, *‘will not confirm or follow a routine… cannot follow one instruction’*.

**CP/HCU and CP/LCU boys’ description of being parented.** Two main themes within the boys’ descriptions of being parented are presented, which illustrate differences and similarities in how boys with CP/HCU and CP/LCU describe their experience of being parented. Both CP/HCU and CP/LCU boys described parental support, with CP/HCU boys describing parental willingness to support in the face of adversity, and CP/LCU describing parental understanding and guidance. Both CP/HCU and CP/CLU boys described parents solely as a provider of basic needs.

***Parental support.***

***Theme 1: Parental willingness to support in the face of adversity.***

CP/HCU boys seemed to acknowledge parental support was there for them even when things were very difficult, or their behaviour did not warrant such support. One CP/HCU boy reported, *‘If I get arrested, she’ll come get me’*, and another commented, *‘it didn’t matter how I treated her, she was always nice to me’*. Another CP/HCU boy noted his parent’s optimism in the face of challenges, ‘*always being happy and trying not to ever be unpositive (sic), always looking on the bright side’*.

***Theme 2: Parental understanding.***

CP/LCU boys described their parents as having them in mind. One CP/LCU boy described how his parent, *‘texts me to see where and how I am’*, and another reported that his parent *‘thinks about me’*. CP/LCU boys also reported feeling understood by their parent/caregiver. One CP/LCU boy reported that his parent, *‘understands my perspective’*, and another remarked, ‘*she understands me and now I realise how well she has raised me’*.

***Theme 3: Parental guidance.***

CP/LCU boys seemed keenly aware of their parent’s attempts to keep them on the right path. Several CP/LCU boys remarked about parental guidance with statements such as, *‘If I do something wrong, I am usually spoken to; If I do something right I am praised’*, and, *‘She takes care of me by making sure that before I do or say something I understand it. She also takes care of by ensuring I know what is right and wrong and also what is and isn't acceptable’*, as well as, *‘…points out correct (sic) or errors with my potential views’.* One CP/LCU boy commented that his parent would, *‘tell me what to do or show me’*, and another boy remarked that his parent would *‘help me come to my own decisions about issues/problems’*.

***Parent as a provider of basic needs.***

When asked to describe their experience of being parented, a considerable number of CP/HCU (31%) and CP/LCU (22%) boys described their caregiver solely as a provider of basic needs with no mention of any emotional support or affection. These descriptions were focussed exclusively on the very basic structures of caregiving, such as, *‘she pays the electric bills’*, *‘cooks and cleans’*, *‘gives me shelter, a room to sleep in’*, and *‘she gives me food, she dresses me, she pays for my house bills’*. The absence of emotional descriptions occurred very infrequently in TD boys’ descriptions of caregiving (8%).
